# Commentary: Editorial: Strengthening food labeling policies in Brazil

**DOI:** 10.3389/fnut.2023.1331250

**Published:** 2023-12-08

**Authors:** Bruna Eugênia Ferreira Mota, Iasmim Cristiane de Alcântara, Perciliany Martins de Souza, Gabriela Guerra Leal Souza, Laís Amaral Mais, Camila Carvalho Menezes, Isabel Antunes David

**Affiliations:** ^1^Laboratory of Psychophysiology, Institute of Exact and Biological Sciences, Department of Biological Sciences, Federal University of Ouro Preto, Ouro Preto, Brazil; ^2^Healthy and Sustainable Diets Program, Brazilian Institute for Consumer Defense (IDEC), São Paulo, Brazil; ^3^Department of Food, School of Nutrition, Federal University of Ouro Preto, Ouro Preto, Brazil; ^4^Laboratory of Neurophysiology of Behavior, Department of Physiology and Pharmacology, Biomedical Institute, Federal University Fluminense, Niterói, Brazil

**Keywords:** front-of-pack nutrition labeling, ultra-processed foods, Brazil, Latin America, food policy

## 1 Introduction

The research topic “Strengthening Food Labeling Policies in Brazil” provided an overview of food labeling policies in Brazil. Regarding the implementation of front-of-package nutrition labeling (FoPNL), the results discussed in the research topic suggest that the effectiveness of FoPNL systems depends on a number of factors including the design of the label, see for example, Prates et al. (1), Fernandes et al. (2), and Scapin et al. (3); and the nutrient profile model used to define which products are subject to them, see for example, Borges et al. (4) and Tomaz et al. (5). Thus, Prates et al. (1) showed that the Brazilian magnifying glass model did not perform as well as the triangular or octagonal FoPNL models, while Borges et al. (4) showed that because of the nutrient profile model adopted in Brazil, which is considerably more permissive than the nutrient profile model of the Pan-American Health Organization (PAHO), fewer products will receive FoPNL in Brazil.

The aim of this commentary is to extend the discussion on the new Brazilian FoPNL regulations. Here, we will briefly comment on how Brazil missed an opportunity to make a more significant public health advance during the implementation of FoPNL by failing to learn from the experience of other Latin American countries.

## 2 Front-of-package nutrition labeling in Latin America: where does Brazil stand?

**Figure 1:**
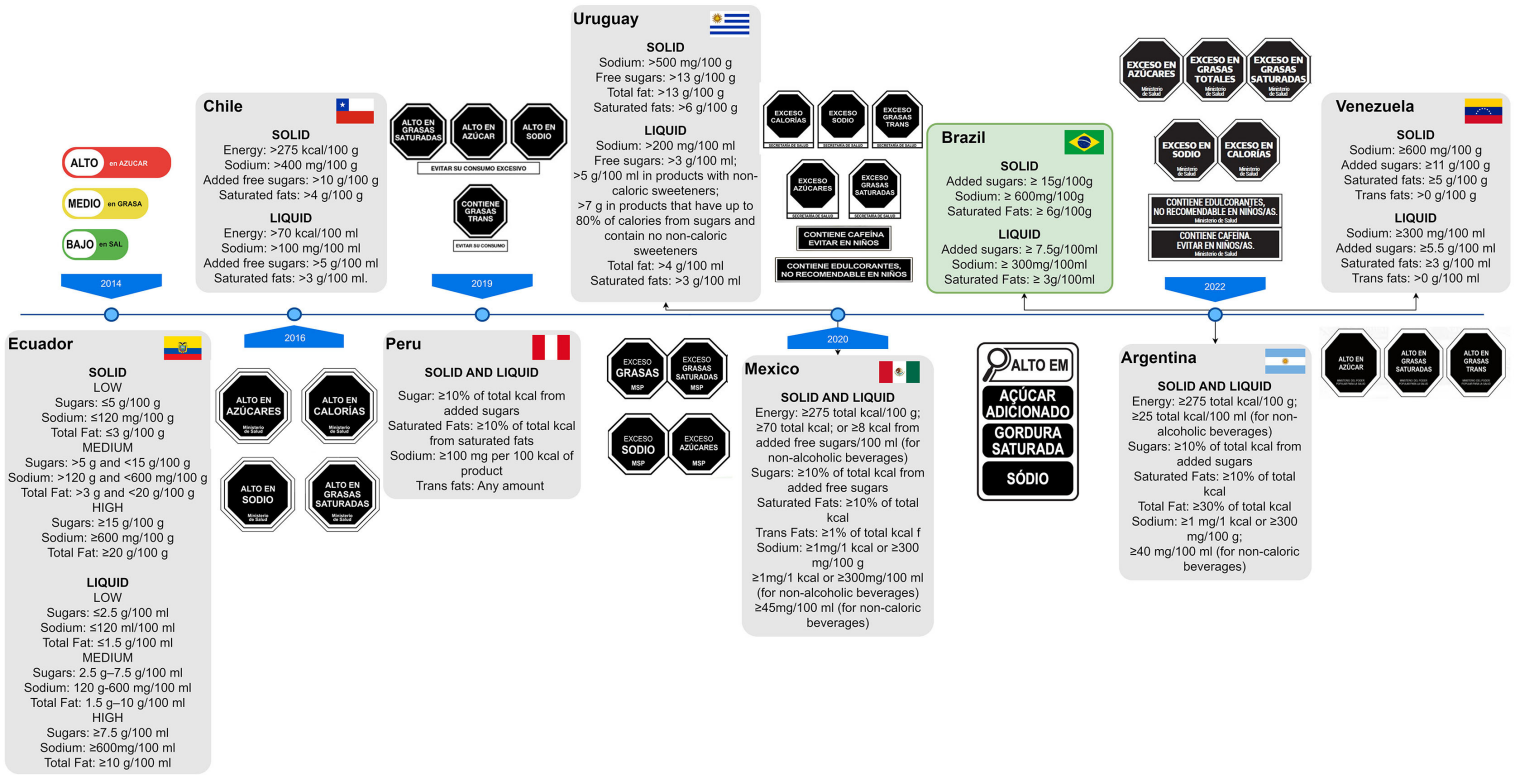
Timeline of regulations for front-of-pack nutrition labeling (FoPNL) implementation and specifications in Latin American countries. Brazil was one of the latest countries to implement the FoPNL, although it was one of the first to start its discussion. If the country's government agreed to roll out FoPNL in stages, the nutrient threshold is relative to the most recent stage. The data were taken from Crosbie et al. (24).

Of the Latin American countries that have implemented FoPNL, six have adopted black octagons, similar to stop signs (Chile, Peru, Uruguay, Mexico, Argentina, and Venezuela) (Figure 1). This design has been shown to have a positive impact on consumers, allowing them to make more informed decisions (6–9).

The implementation of FoPNL in many Latin American countries occurred with modifications to the proposed FoPNL based on previous experience and scientific evidence relating to the use of the octagon model and the accompanying text in these countries. Chile (10) was the first country to adopt “high in” octagons as a warning label. Peru modified the Chilean octagons, adding a text below them to discourage the consumption of foods with excess nutrients of concern (6). Uruguay then changed the wording inside the octagon from “high in” to “excess” (7), Mexico did the same, and added text warnings below the octagons about the presence of caffeine and non-sugar sweeteners (NSS), recommending that they should be avoided by children (11). Argentina then followed suit, implementing the octagons featuring the word “excess,” and adding text warnings in respect of caffeine and NSS (12). It is important to note that these warnings regarding NSS were adopted before the official recommendation against their consumption was issued from the World Health Organization (WHO) (13). However, in Mexico and Argentina regulations, it was considered findings showing that the Chilean food industry has increased the use of food additives in its products to avoid adding sugar warnings as a FoPNL, exposing the population, especially children, to the increased consumption of these substances (14).

Therefore, most Latin American countries promoted modifications and improvements in FoPNL regulation based on the experiences of other countries and complemented the FoPNL regulations with other associated regulatory measures, such as the regulation of marketing making health claims and the restriction of the sale of some products in schools (11, 12, 15). However, Brazil did not seem to follow other Latin American countries by learning from their experiences or scientific evidence, and decided to use a magnifying glass (see Figure 1).

## 3 Discussion

A randomized study carried out in Brazil by Khandpur et al. (16) found that the most effective FoPNL model was a black triangular warning, with the words “high in” in white letters, which would be related to excess sodium, sugar, total fats, and saturated fats, in addition to the presence of sweeteners and trans fats (16). The study found that this model made the warning more visible to consumers, captured their attention, and informed them about the nutritional content of the food. Another study by Khandpur et al. (17) compared the magnifying glass, which was the model chosen by the Brazilian Health Regulatory Agency (*Agência Nacional de Vigilância Sanitária*, Anvisa) (18), and the black triangle FoPNL models. They found that the triangle was better than the magnifying glass for helping participants to identify healthier products. It is important to note that the magnifying glass was incorporated into the Brazilian regulations, without any scientific evidence that it would perform better than other FoPNL models (19).

In addition, Brazil did not adopt the PAHO nutrient profile model, proposed in 2016, whose use is recommended when using a FoPNL (20). Mexico, in early 2020, was the first country in Latin America to implement PAHO's nutrient profile model, and Argentina and Peru also recently adopted these criteria, while Chile and Ecuador established their own classification criteria, as their regulation preceded the release of the PAHO nutrient profile model (21). Although Brazil implemented FoPNL in 2020, years after the establishment of PAHO's nutrient profile model, it was not adopted alongside the FoPNL legislation. Duran et al. (21) showed that 63% of packaged food products would feature a FoPNL if the PAHO nutrient profile model was considered in Brazil. However, only 45% of the products would feature an FoPNL even if the strictest thresholds proposed by Anvisa at that point were applied. However, the adopted thresholds were less stringent. Therefore, in contrast to other Latin American countries, the nutrient thresholds approved in Brazil's nutrition labeling regulation to define which products would feature the magnifying glass do not capture all products that exceed the recommended nutrient intake goals stipulated by the PAHO nutrient profile model.

Brazil could have been among the first countries in Latin America to adopt an FoPNL system; however, it took 6 years (from 2014 to 2020) for Anvisa to approve the new labeling regulations for Brazil. Although there is currently a lack of understanding about the role of the food industry in this process, Mais et al. (22) suggested that the food industry may have attempted to delegitimize the implementation of FoPNL in Brazil, leading to delays. The authors also highlighted that the food industry plays an important role in the Brazilian economy, which may have encouraged the government to make less stringent decisions regarding food labeling (22).

It should be noted that the Brazilian FoPNL have some strengths, especially in respect of the nutrition facts panel, which include mandatory information on total and added sugars and nutritional information per 100 g (or ml), as well as improved ease of understanding resulting from better design (23). However, considering the abundant scientific evidence existing prior to the introduction of the regulations, Brazil lost the opportunity to create FoPNL regulations that produced the maximum benefit for its population. The experiences of other Latin American countries should be used to improve the Brazilian regulations, and to incorporate other measures linked to FoPNL in order to better address issues related to consumer food choices, and, thereby, improve the nutrition and health of the Brazilian population.

## Author contributions

BM: Conceptualization, Investigation, Methodology, Writing – original draft. IA: Investigation, Methodology, Writing – original draft. PS: Investigation, Methodology, Writing – review & editing. GS: Funding acquisition, Project administration, Supervision, Writing – review & editing. LM: Writing – review & editing, Investigation, Validation, Visualization. CM: Methodology, Writing – review & editing, Conceptualization, Supervision. ID: Funding acquisition, Investigation, Methodology, Project administration, Software, Validation, Visualization, Writing – review & editing.
